# Evaluation of Profile Control and Oil Displacement Effect of Starch Gel and Nano-MoS_2_ Combination System in High-Temperature Heterogeneous Reservoir

**DOI:** 10.3390/gels10020127

**Published:** 2024-02-04

**Authors:** Lianfeng Zhang, Yanhua Liu, Zhengxin Wang, Hao Li, Yuheng Zhao, Yinuo Pan, Yang Liu, Weifeng Yuan, Jirui Hou

**Affiliations:** 1 Key Laboratory of Enhanced Oil Recovery of Henan Province, Nanyang 473000, China; zhlf1109@126.com (L.Z.); yjycsllyh.hnyt@sinopec.com (Y.L.); yjycslwzx.hnyt@sinopec.com (Z.W.); lih7327.hnyt@sinopec.com (H.L.); 2 Exploration and Development Research Institute of Henan Oilfield Branch Company, Sinopec, Nanyang 473000, China; 3 Research Institute of Unconventional Petroleum Science and Technology, China University of Petroleum (Beijing), Beijing 102249, China; z13633831585@163.com (Y.Z.); 2022211726@student.cup.edu.cn (Y.P.); 2022216642@student.cup.edu.cn (Y.L.)

**Keywords:** starch gel, nano-MoS_2_, profile control and oil displacement, enhanced oil recovery, high-temperature heterogeneous reservoirs

## Abstract

The Henan Oilfield’s medium-permeability blocks face challenges such as high temperatures and severe heterogeneity, making conventional flooding systems less effective. The starch gel system is an efficient approach for deep profile control in high-temperature reservoirs, while the nano-MoS_2_ system is a promising enhanced oil recovery (EOR) technology for high-temperature low-permeability reservoirs. Combining these two may achieve the dual effects of profile control and oil displacement, significantly enhancing oil recovery in high-temperature heterogeneous reservoirs. The basic performance evaluation of the combination system was carried out under reservoir temperature. Displacement experiments were conducted in target blocks under different permeabilities and extreme disparity core flooding to evaluate the combination system’s oil displacement effect. Additionally, the displacement effects and mechanisms of the starch gel and nano-MoS_2_ combination system in heterogeneous reservoirs were evaluated by simulating interlayer and intralayer heterogeneity models. The results show that the single nano-MoS_2_ system’s efficiency decreases with increased core permeability, and its effectiveness is limited in triple and quintuple disparity parallel experiments. After injecting the starch gel–nano-MoS_2_ combination system, the enhanced oil recovery effect was significant. The interlayer and intralayer heterogeneous models demonstrated that the primary water flooding mainly affected the high-permeability layers, while the starch gel effectively blocked the dominant channels, forcing the nano-MoS_2_ oil displacement system towards unswept areas. This coordination significantly enhanced oil displacement, with the combination system improving recovery by 15.33 and 12.20 percentage points, respectively. This research indicates that the starch gel and nano-MoS_2_ combination flooding technique holds promise for enhancing oil recovery in high-temperature heterogeneous reservoirs of Henan Oilfield, providing foundational support for field applications.

## 1. Introduction

Due to reservoir heterogeneity and long-term water flooding, injected water tends to flow along preferential channels, leading to issues like water flooding in production wells and ineffective circulation between injection and production wells [1,2,3]. This makes it challenging to mobilize the oil in the reservoir effectively. Reservoir heterogeneity is one of the key factors affecting the efficiency of water flooding and subsequent enhanced oil recovery efforts [4,5,6]. Interlayer heterogeneity refers to significant permeability differences between individual layers, varying by several times, tens of times, or even hundreds of times [7,8]. These permeability differences cause the injected water to rapidly advance through layers with good connectivity and high permeability, leading to early water breakthrough in production wells and rapid increases in water cut, even resulting in water flooding and shutdown. Meanwhile, significant residual oil remains in the low-permeability layers, highlighting the contrast between layers. Intralayer heterogeneity refers to differences in reservoir properties within a single sand layer [9,10]. For instance, in rhythmically deposited reservoirs, the bottom high-permeability segments are prone to intralayer advancement, while the top low-permeability segments accumulate residual oil, emphasizing the intralayer contrast.

In response to the contradictions of interlayer and intralayer water flooding in reservoirs, profile modification and plugging agents, like polymers, frozen gels, gels, and particulates, have shown effectiveness in improving reservoir heterogeneity, with good field application results [11,12,13]. However, polymers and conventional frozen gel water plugging agents tend to degrade and lose effectiveness in high-temperature reservoirs, leading to a decrease in strength and failing to meet plugging requirements [14,15]. Gel systems block formations by swelling upon water absorption, but the gelation time is influenced by many factors, and the plugging measures become ineffective once water breaks through the gel layer, reducing the profile modification effect [16,17]. Polymer/nano-microsphere systems need to be selected based on the actual pore sizes of the reservoir, and their plugging effectiveness in high-permeability channels is limited [18,19,20].

In contrast, starch gels can address these issues effectively. Previous research has demonstrated that starch gel systems exhibit strong plugging effects. Tang et al. [21] introduced starch graft copolymer plugging agents as a novel, efficient, and cost-effective solution for water plugging. Laboratory experiments demonstrated that these starch strong gel plugging agents possess selective plugging capabilities and achieve excellent plugging results. Furthermore, field tests of modified starch strong gel plugging agents were conducted in well N12-P6 in the JIDONG Oilfield. The field test results indicated that prior to implementation, well N12-P6 had a water cut as high as 98%, but after implementation, the daily water cut significantly decreased, reaching below 45%. The effective production increase period exceeded one year, indicating the long-term stability of the starch gel. In addressing the issue of CO_2_ gas channeling, Zhao et al. [22] proposed the use of ethylenediamine and modified starch gel as plugging agents to plug the gas channels. Laboratory experiments demonstrated that the injection of modified starch gel and ethylenediamine rapidly increased injection pressure, effectively plugging high-capacity gas channels and fractures. Building on the research by Zhao et al., Hao et al. [23] further evaluated the static and plugging performance of gel systems. They identified a high-strength starch gel composed of 8% modified starch + acrylamide, 0.05% crosslinker, 0.15% initiator, and 0.15% stabilizer as an optimal plugging agent. Under acidic conditions, this gel effectively sealed fractures, leading to a rapid decrease in water cut, reaching a minimum of 29%. The displacement pressure drop sharply increased from 64.525 psi to over 145 psi, indicating complete fracture plugging and forcing the injected gas into lower-permeability rock layers.

The primary characteristics of modified starch gel plugging agents are as follows: Before gelation, starch gels are purely viscous fluids, with a pressure index that linearly increases with the injection volume and exhibits a small increase in amplitude and ease of injection. After gelation, they transform into viscoelastic fluids with strong plugging capabilities [22]. Luo et al. [24] developed an in situ starch grafted copolymer gel system (ISGCG) as an effective plugging material and systematically evaluated its gelation properties. The experimental results showed that the ISGCG system exhibits shear thinning behavior before gelation. The higher the shear rate, the more orderly the alignment of modified starch molecules along the shear flow direction, resulting in lower flow resistance and, consequently, lower viscosity. Notably, at a shear rate of 7.34 s^−1^, the viscosity of the gelant is only 107 mPa·s, demonstrating its excellent injectability in formations. Furthermore, the gelation time can be controlled within 4–8 h, indicating very good pumping performance. Additionally, Luo et al. [24] incorporated sand grains of various sizes into modified starch gels. All of them can be completely crosslinked, and the sand particles can be cemented in the gel as a whole and have strong gel strength. Furthermore, following the completion of sand column plugging experiments, the gel system can ultimately cement the gravel together, making the gravels form a larger overall structure. This demonstrated the modified starch gel system’s ability to form strong gels within subsurface pore structures, exhibiting commendable adhesion to rocks and sand grains. Finally, the ISGCG was successfully applied in the Xinjiang oilfield, where the daily water cut of the treated wells decreased from 92.7% to 75.8%, and daily oil production increased from 8.1 to 18.8 t/d, signifying remarkable effectiveness.

Furthermore, Leng et al. [25] systematically compared the deep profile control effects of modified starch gels with polymer gels in sandstone reservoirs. Their study revealed that the injectability and plugging properties of modified starch gels are superior to those of viscoelastic polymer gels. During the gel flooding stage, the modified starch gels maintained their rigid shape, unlike the deformation and migration behavior observed with polymer gels. In the subsequent water flooding stage, the modified starch gels remained immobile, effectively sealing high-permeability layers and enhancing the sweep efficiency in low-permeability layers by 60.00%. In contrast, polymer gels continued to flow through the core, increasing the sweep efficiency in low-permeability layers by 37.26%.

In a word, starch gels have moderate viscosity, ease of injection, high underground gelation strength, strong adhesion to rock, and long-term stability, effectively plugging high-strength leakage channels. However, when using starch gel alone for profile modification, the subsequent water flooding oil washing capability is generally limited, only moderately improving oil recovery in heterogeneous reservoirs.

Combining starch gel with oil displacement agents is expected to achieve the dual effects of profile control and oil displacement, significantly enhancing oil recovery in heterogeneous reservoirs. Researchers like Zhao et al. [22] and Hao et al. [23] used starch gel systems to plug escaping reservoirs and conducted subsequent displacement with CO_2_. Their results showed that the gelation time of the starch gel system is controllable, with good injection performance and appropriate plugging strength, making it feasible for field implementation. Li et al. [26] combined dispersed particle gel with surfactant for combination profile modification and displacement. Their experiments showed that this combination has been developed as a cost-effective EOR method due to highly beneficial synergistic behavior of improving both sweep efficiency and displacement efficiency. Currently, there are no studies on the combination profile control and oil displacement of starch gel with nanomaterials for high-temperature heterogeneous reservoirs.

Nano-displacement technology is a highly promising enhanced oil recovery technique for high-temperature, low-permeability, or ultra-low-permeability reservoirs. Modified nanomaterials maintain good stability in high-temperature, high-salinity environments and have certain flow control capabilities [27,28,29]. Nanomaterials reported to enhance oil recovery include SiO_2_ [30], TiO_2_ [31], Al_2_O_3_ [32], CuO [33], ZnO [34], and graphene [35]. Nanofluids, created by dispersing these nanomaterials in specific solvents, enhance oil recovery by reducing oil–water interfacial tension [36], altering rock surface wettability [37], forming in situ Pickering emulsions [38], creating structural disjoining pressure (SDP) [39], and reducing oil viscosity [40]. Nano-MoS_2_ is a flexible, plate-like material with nano-scale dimensions, approximately 60 nm×80 nm in size and an average thickness of 1.2 nm [41]. Amphiphilic nanosheets, especially, exhibit higher interfacial activity compared to spherical and rod-shaped nanomaterials [42,43]. Consequently, these amphiphilic nanosheets demonstrate greater potential in reducing interfacial tension (IFT), stabilizing emulsions, and altering interfacial properties. Furthermore, compared with the SDP formation when using spherical nanoparticles (20 vol%), Qu et al. [44] proposed that far fewer nanosheets (0.005 wt%) are needed to generate SDP.

Infant Raj et al. [41] previously demonstrated the potential of two-dimensional molybdenum disulfide (MoS_2_) nanosheets in enhancing crude oil recovery. The synthesized amphiphilic MoS_2_ nanosheets, even at extremely low concentrations (0.005 wt%), increased recovery by 18.25% in 25 mD permeability cores saturated with crude oil. Liang et al. [45] investigated the mechanisms of enhanced recovery by modified MoS_2_ nanosheets, focusing on interfacial tension reduction, wettability alteration, and emulsion stabilization. The results show that ultra-low-concentration MoS_2_ nanofluid (50 mg/L) can decrease the IFT to 2.6 mN/m, change the contact angle (CTA) from 131.2° to 51.7°, and significantly enhance emulsion stability. Similarly, ultra-low-concentration MoS_2_ nanofluids could increase oil displacement efficiency by 14% after water flooding. Qu et al. [44] synthesized amphiphilic MoS_2_ nanosheets through a one-step simple hydrothermal method and conducted a comprehensive study of their physicochemical properties. The results showed that these nanosheets, with a distinctly ultra-thin lamellar structure, could stably disperse in water at ultra-low concentrations (50 mg/L), reducing oil–water interfacial tension, altering solid surface wettability, and stabilizing emulsions. Further, Ming Qu et al. [46] undertook both laboratory studies and field applications of amphiphilic MoS_2_ nanosheets for enhanced oil recovery. Following the injection of ODA-MoS_2_ nanofluid, oil production notably increased from 0.8 t/d to 1.4 t/d, while the water cut decreased significantly from 88% to 78.8%. The MoS_2_ oil displacement system displays excellent dispersion stability and robust temperature resistance. Previous studies have produced amphiphilic nano-MoS_2_ with good dispersion stability through chemical modification [44,45,46]. Liang et al. [47] reported that nano-MoS_2_ solutions were applied in enhanced oil recovery tests at the Shengli Oilfield (Shandong, China) Xin 154 well group (reservoir temperature 114 °C) and the Tahe Oilfield (Xinjiang, China) TK7-459 well group (reservoir temperature 130 °C), demonstrating a significant oil production increase and affirming the exceptional thermal stability of MoS_2_. Numerous laboratory studies and field applications have demonstrated that nano-MoS_2_ oil displacement technology is an effective successor technology for enhancing oil recovery in high water-cut reservoirs or after chemical flooding, significantly increasing oil recovery [44,45,46,47,48].

In light of previous studies, MoS_2_ nanosheets have been selected for recovery enhancement. Firstly, MoS_2_ nanosheets, being lamellar nanomaterials with a 2 nm thickness and flexible properties, possess a thinner profile and greater deformability compared to spherical nanoparticles with larger diameters and rigidity. This makes them more effective in penetrating nanoscale pores and throats. Secondly, MoS_2_ nanosheets, unlike other lamellar nanomaterials such as graphite, can be synthesized through a simple, direct hydrothermal method, making them suitable for industrial production. Finally, the concentration of MoS_2_ used is 0.005 wt% to 0.01 wt%, significantly lower than other nanofluids.

However, due to characteristics like low viscosity, nano-MoS_2_ has difficulty effectively increasing the sweep efficiency in heterogeneous formations. If nano-MoS_2_ is used for oil displacement without profile modification, its flow control capabilities may not meet the requirements in reservoirs with strong heterogeneity and high permeability, leading to leakage and affecting the nano-MoS_2_’s oil recovery effect while increasing usage costs. Starch gel is an effective means of deep reservoir plugging. Combining starch gel with nano-MoS_2_ is expected to achieve the dual effects of profile control and oil displacement, significantly improving sweep efficiency and oil displacement efficiency in heterogeneous reservoirs.

The Anpeng Block in Henan Oilfield exhibits significant heterogeneity, with oil sand body permeabilities ranging from 40 × 10^−3^ μm^2^ to 453 × 10^−3^ μm^2^, averaging 168 × 10^−3^ μm^2^, and an average porosity of 15.3%. The reservoir temperature ranges from 91.1 °C to 95.0 °C.

In response to the high temperatures and severe heterogeneity in the medium-permeability blocks of the Henan Oilfield’s Anpeng area, a study was conducted on the adaptability of the starch gel and nano-MoS_2_ combination system in high-temperature medium-permeability reservoirs. Firstly, the basic properties of the starch gel and nano-MoS_2_ combination system at high temperature were evaluated. Secondly, experiments were conducted under reservoir temperatures of 95 °C with different permeabilities and parallel core flooding to evaluate the displacement effect of the starch gel and nano-MoS_2_ combination system. Finally, using the interlayer and intralayer heterogeneous models, the profile modification effect of the combination system in interlayer or intralayer heterogeneous reservoirs was assessed, clarifying the adaptability of the starch gel and nano-MoS_2_ combination profile control and oil displacement system in high-temperature reservoirs.

## 2. Results and Discussion

### 2.1. Evaluation of Basic Properties of Starch Gel and Nano-MoS_2_

**Figure 1:**
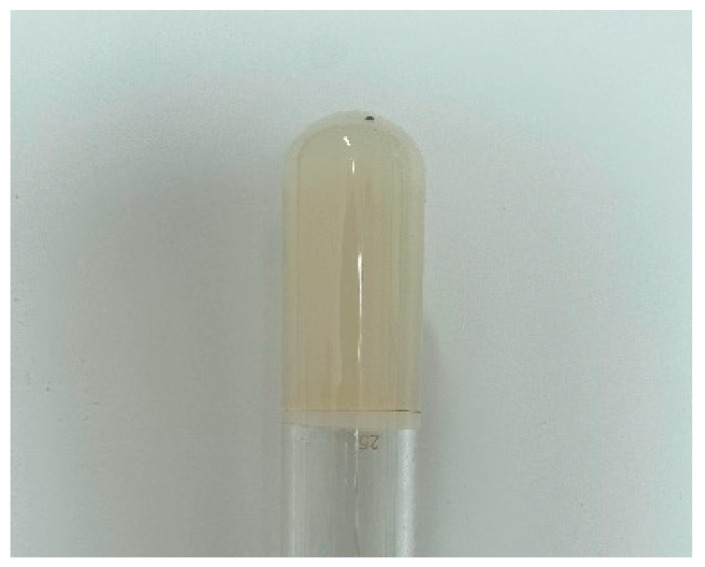
Appearance of starch gel after gelation at 95 °C.

A certain amount of modified starch, acrylamide monomer, crosslinker, and initiator was proportionately taken to prepare a starch gel solution. The starch gel solution was placed in a constant temperature oven set at 95 °C, and the gelation time and gel strength of the starch gel solution were recorded. Experimental results show that the gelation time of the starch gel is 10~12 h, and its gel strength reaches the H level (Sydansk’s Gel Strength Code, GSC) [49,50], as shown in Figure 1. This indicates that the prepared starch gel has good temperature resistance and can stably form gel at 95 °C, with rigid gel strength.

Previous studies have shown that the oil displacement system of MoS_2_ exhibits excellent dispersion stability [44,45,46] and good temperature resistance [47]. It also produces structuring disjoining pressure at ultra-low concentrations [44], altering wettability [44,45,46], reducing interfacial tension, and emulsifying crude oil, among other functions [47].

**Figure 2:**
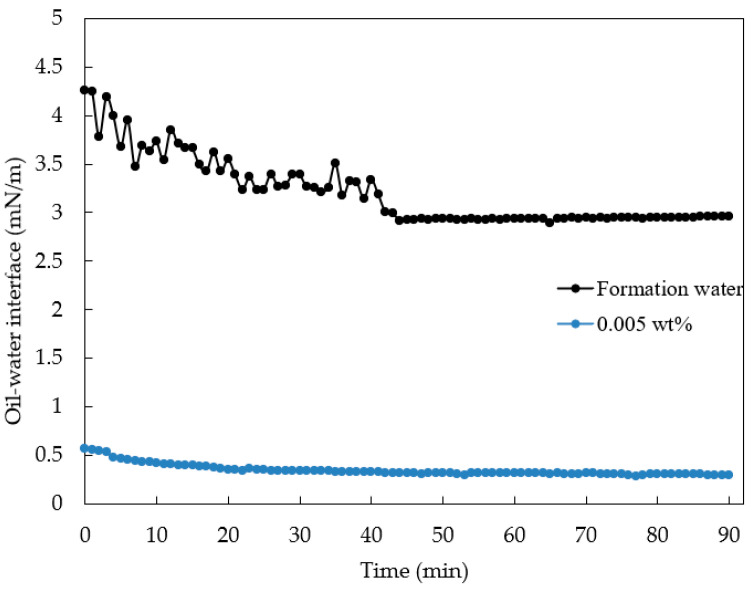
Tension–time curve of oil–water interface.

**Figure 3:**
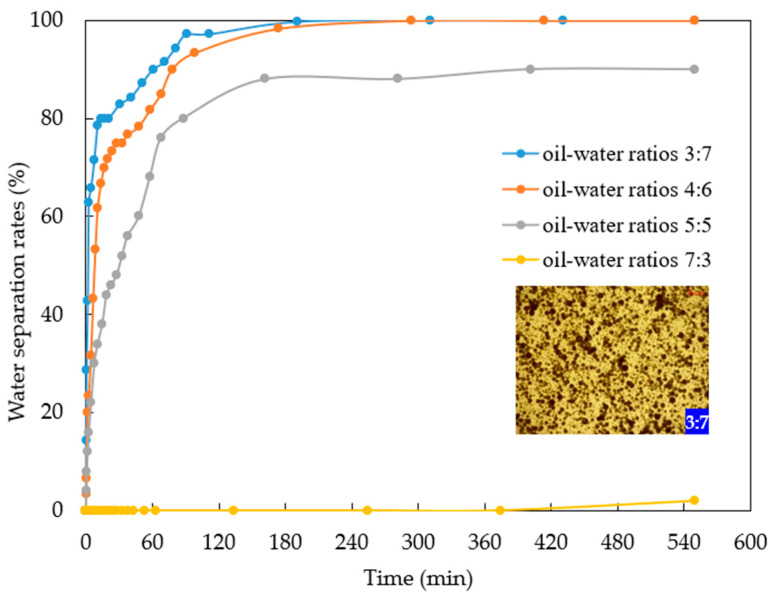
The water separation rate of emulsion.

Based on previous research, preliminary assessments of its compatibility with Henan oilfield crude oil were conducted. The measurement results at 95 °C show that the oil–water interfacial tension in formation water is 2.955 mN/m, which could be the result of a combination of temperature, formation water, and crude oil properties [51,52,53]. And a 0.005% concentration of nano-MoS_2_ can reduce the oil–water interfacial tension to 0.297 mN/m (as shown in Figure 2). Furthermore, the system exhibited a good emulsification capacity for crude oil, consistently forming O/W emulsions across varying oil–water ratios. Emulsions with oil–water ratios of 3:7, 4:6, and 5:5 demonstrate water separation rates of 100%, 100%, and 90%, respectively (see Figure 3). Notably, at an oil–water ratio of 7:3, a stable O/W-type emulsion is formed without visible water phase separation or significant stratification. This shows that the nano-MoS_2_ solution has the ability of emulsification and rapid demulsification, which avoids the difficult problem of oil–water demulsification in the produced solution. This is a characteristic of nanofluids. Nanoparticles can improve emulsion stability by forming single- or multilayered nanoparticle interfacial films at the oil–water interface [54,55,56]. Therefore, the nano-MoS_2_ solution has better emulsification ability than water [57]. However, the viscosity of the nanofluid is the same as that of saline, so when the shear action stops, the emulsion is broken more quickly than that of surfactant emulsions.

Basic performance evaluation experiments demonstrate that nano-MoS_2_ possesses commendable thermal resistance. Even at high temperatures of 95 °C, it retains its functionality in reducing interfacial tension and emulsifying crude oil. Preliminary results suggest that the combination system of starch gel and nano-MoS_2_ is adaptable to reservoir temperatures and the properties of crude oil. This compatibility was further explored through displacement experiments assessing the oil displacement efficiency of the starch gel and nano-MoS_2_ system.

### 2.2. Evaluation of Oil Displacement Efficiency of Nano-MoS_2_ under Different Core Permeabilities

To verify the oil displacement effect of the nano-MoS_2_ system, considering economic factors and past field application experiences, a system with a mass concentration of 0.005% nano-MoS_2_ was selected. Experiments were conducted at a reservoir temperature of 95 °C to evaluate the oil displacement efficiency within the target permeability range of the reservoir. The results under different permeabilities (air-measured permeabilities of 100, 200, 300, and 500 mD) are presented in Table 1.

From Table 1, it is evident that the nano-MoS_2_ system significantly enhances oil recovery in the cores of different permeabilities. Specifically, the improvements in oil recovery were 18.03%, 20.06%, 14.52%, and 12.31% for cores with permeabilities of 100 mD, 200 mD, 300 mD, and 500 mD, respectively. These results indicate that within a certain range, as core permeability increases, the nano-MoS_2_ system penetrates more pore throats, activating oil in areas not reached by primary water flooding. This enhances sweep efficiency and washing efficiency. However, as permeability further increases, the nano-MoS_2_ system tends to channel through preferential pathways, reducing sweep efficiency and leading to a decrease in oil recovery with increasing permeability.

### 2.3. Evaluation of Combination System Oil Displacement Efficiency under Different Heterogeneity Extents Using a Parallel Model

**Figure 4:**
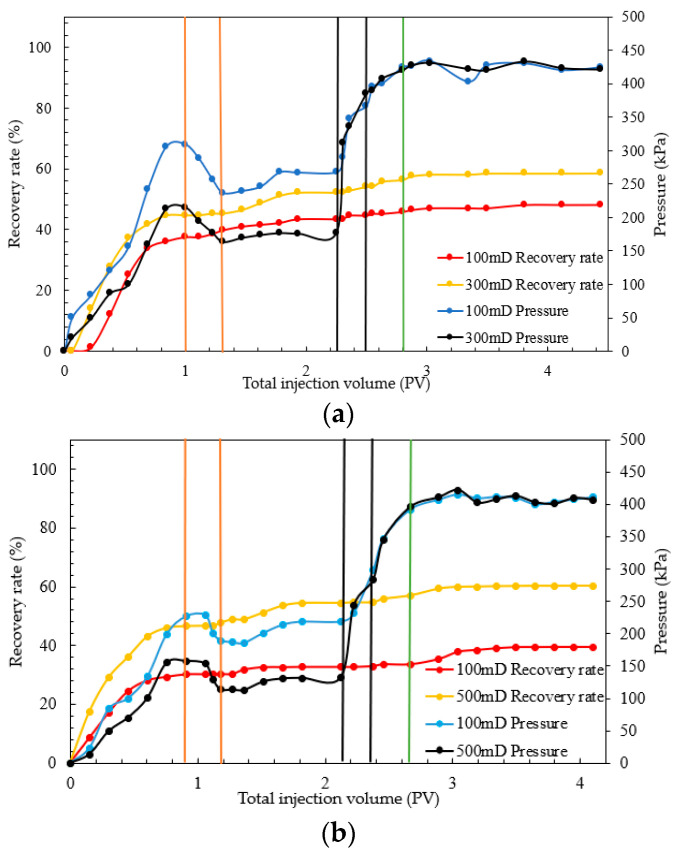
Experimental results of parallel model combination system: (**a**) threefold; (**b**) fivefold.

**Figure 5:**
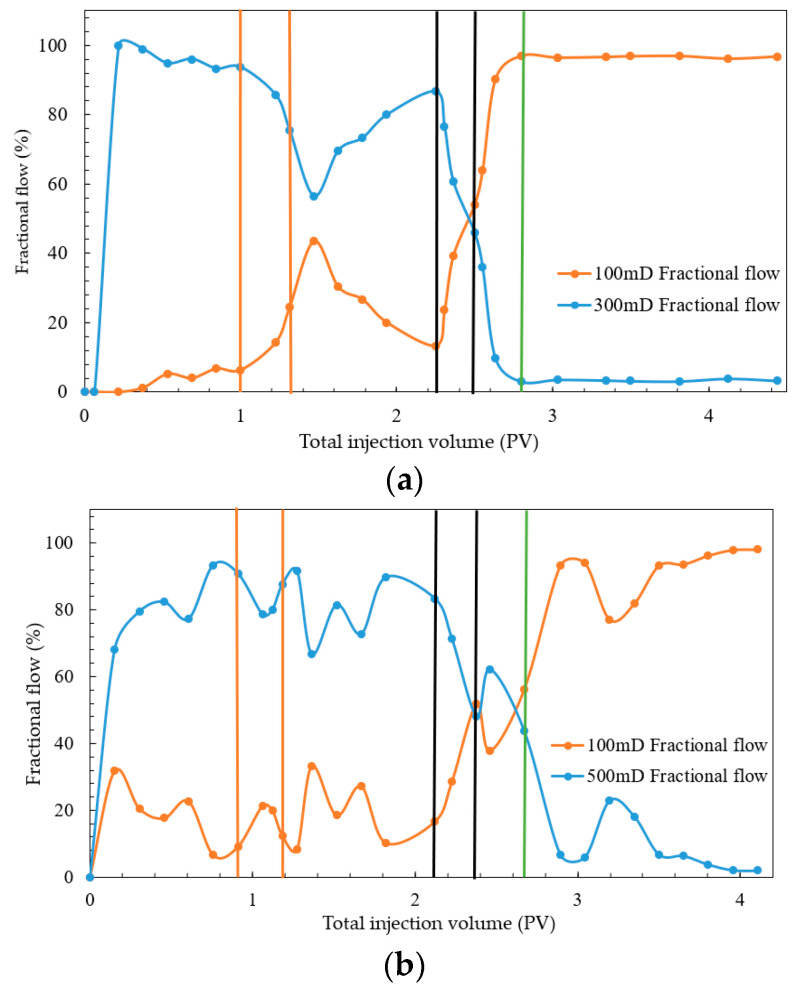
Fractional flow of oil displacement experiment of combination system with parallel mode: (**a**) threefold; (**b**) fivefold.

To validate the oil displacement effect of a combination system of starch gel and nano-MoS_2_ in heterogeneous reservoirs and compare it with the use of nano-MoS_2_ alone, cores with air-measured permeabilities of 100 mD, 300 mD, and 500 mD were selected. Experiments were conducted under reservoir conditions at 95 °C to evaluate the displacement efficiency of the combination system in parallel models with threefold (100 mD and 300 mD) and fivefold (100 mD and 500 mD) heterogeneity extents. The results are shown in Figure 4 and Figure 5 and Table 2.

From Figure 4 and Table 2, during the water flooding phase, cores with higher permeability showed a rapid increase in oil recovery, with higher water flooding recovery rates than those in lower-permeability cores. In the nano-MoS_2_ phase, the oil recovery in the threefold heterogeneity model increased by 7.44 and 5.90 percentage points for high- and relatively low-permeability layers (the term “relatively low-permeability” mentioned here refers to cores with permeability lower than that of 300 mD or 500 mD cores, and the following applies), respectively. In the fivefold heterogeneity model, the increases were 7.80 and 2.48 percentage points. This suggests that the nano-MoS_2_ exhibits a certain degree of profile control and oil displacement. However, with increasing heterogeneity, the improvement in relatively low-permeability layers diminishes.

In the combination displacement phase with starch gel and nano-MoS_2_, significant improvements were observed in both heterogeneity models. In the threefold model, oil recovery increased by 6.34 and 4.72 percentage points in high- and relatively low-permeability layers, respectively; in the fivefold model, the increases were 5.84 and 6.80 percentage points. This indicates that after injection of the starch gel system and a three-day static period, the gel formed within the porous medium effectively plugged preferential channels, altering the flow direction of the subsequent displacement fluids. This allowed the nano-MoS_2_ system to fully interact with the oil in relatively low-permeability cores, thereby enhancing oil recovery.

Furthermore, during the entire displacement process, the injection pressure was initially low, increased slightly during the starch gel phase, and rapidly rose during the nano-MoS_2_ phase, stabilizing after reaching a peak. As shown in Figure 4, during the stable pressure phase of water flooding, the pressures in the high-/relatively low-permeability layers of the threefold and fivefold heterogeneity models were 215.05/308.95 kPa and 165.66/246.80 kPa, respectively. Compared to relatively low-permeability layers, formation water in high-permeability layers during the oil displacement stage tends to flow more easily, leading to channeling phenomena. This results in the formation of preferential pathways in high-permeability layers, causing subsequent displacement fluids to flow through these lower-resistance channels. Consequently, water cut at the outlet increases, oil phase continuity is lost, and difficult-to-recover dispersed residual oil remains trapped in the porous medium. In the combination displacement stage, the injection pressures in the high-/low-permeability layers of the threefold model slowly increased from 176.77/267.49 kPa to 385.22/366.75 kPa, and in the fivefold model, from 132.05/218.59 kPa to 283.57/298.41 kPa, indicating effective plugging of preferential channels by the starch gel.

During fluid injection, due to the differing permeabilities of the two cores, diversion of the injected fluid occurred [58]. The relative flow [59] or fractional flow [60,61] will be higher in the core with higher permeability (the percentage of flow through high-permeability rock cores to the total flow).

Further analysis of the diversion in different heterogeneity parallel cores revealed that during the water flooding stage (Figure 5), diversion in high-permeability cores was predominant. In the nano-MoS_2_ phase, a slight increase in fractional flow in relatively low-permeability cores in the threefold heterogeneity model indicated some autonomous profile control and displacement adjustment by the nano-MoS_2_. The nano-MoS_2_ peeled off the oil film and formed an “oil wall”, changing the displacement phase from water to oil–water phases, thereby increasing the flow resistance of the displacement phase in the porous medium. This led to a reduction in the relative flow in high-permeability cores and an increase in relatively low-permeability cores, forcing the nano-MoS_2_ oil displacement system to turn towards secondary channels for profile control and displacement adjustment. In the fivefold heterogeneity experiment, although a similar self-adjusting phenomenon was observed, its stronger heterogeneity meant that the decrease in the diversion rate in high-permeability cores was small and short-lived. This further indicated that the nano-MoS_2_ exhibits a self-adjusting profile control and displacement effect, albeit limited. In the combination displacement phase, the starch gel entered preferential channels and gelled, causing the subsequent nano-MoS_2_ solution to flow towards relatively low-permeability layers, rapidly increasing the diversion rate in these layers. The experiments showed that after the injection of starch gel, its profile control function weakened the heterogeneity of the parallel cores, improving the displacement efficiency in relatively low-permeability layers.

In conclusion, after primary water flooding, the injection of the nano-MoS_2_ system in the threefold heterogeneity model resulted in significantly higher oil recovery in relatively low-permeability layers compared to the fivefold heterogeneity model, indicating a certain self-adjusting profile control and oil displacement ability of the nano-MoS_2_ solution. The injection of a combination system of starch gel and nano-MoS_2_, with the gel blocking high-permeability layers and forcing the nano-MoS_2_ solution towards relatively low-permeability layers, effectively mobilized oil in unused reservoirs. This suggests that the combination displacement system can effectively enhance oil recovery in strongly heterogeneous reservoirs.

### 2.4. Evaluation of Oil Displacement Efficiency of Starch Gel and Nano-MoS_2_ in Inter- and Intralayer Heterogeneous Model

To further verify the feasibility of the combination displacement technology of starch gel and nano-MoS_2_ in inter- and intralayer heterogeneous reservoirs, and to compare it with the use of the nano-MoS_2_ system alone, displacement experiments were conducted using inter- and intralayer heterogeneity models, simulating one injection and four production wells.

#### 2.4.1. Evaluation of Oil Displacement Effectiveness in Intralayer Heterogeneous Reservoirs

**Figure 6:**
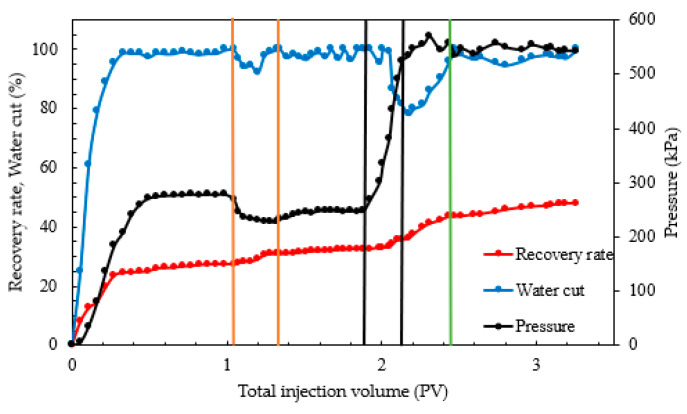
Results of intralayer heterogeneous displacement experiment.

For the intralayer heterogeneous model, the total production of water and oil in three layers was measured, and the overall oil recovery was calculated to analyze the displacement effects of each system. The results of the intralayer heterogeneous displacement experiments are presented in Figure 6 and Table 3.

From Table 3 and Figure 6, it is observed that the oil recovery rate during primary water flooding in the intralayer heterogeneous model was 27.47%, which is relatively low and led to the formation of preferential channels. When the nano-MoS_2_ system was used alone, the oil recovery increased by only 5.07 percentage points, primarily due to the nano-MoS_2_ through preferential pathways. During the combination displacement phase, the water cut decreased to about 75%, and the oil recovery increased by 15.33 percentage points, significantly higher than with the nano-MoS_2_ system alone. The starch gel successfully entered high-permeability layers or preferential channels in the combination system, plugging water breakthrough channels and improving sweep efficiency. This forced the nano-MoS_2_ system towards uninvaded areas, enhancing oil displacement efficiency.

#### 2.4.2. Evaluation of Oil Displacement Effectiveness in Interlayer Heterogeneous Reservoirs

**Figure 7:**
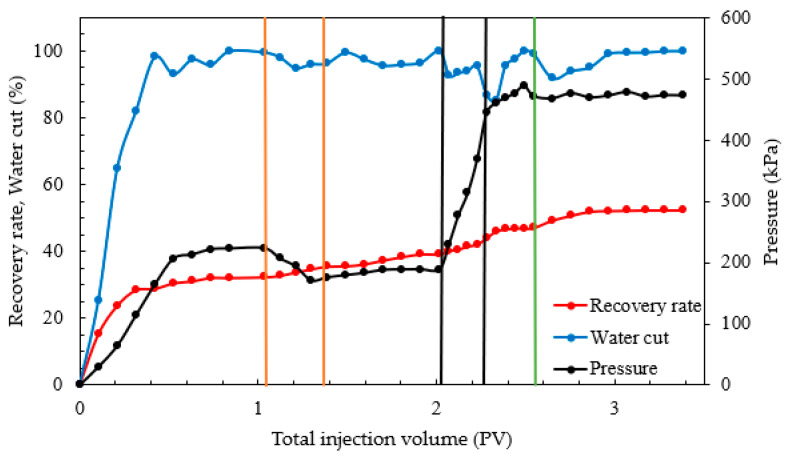
Results of interlayer heterogeneous displacement experiment.

The interlayer heterogeneous model enabled the separate measurement of water and oil production in relatively low-, medium-, and high-permeability layers, as well as the analysis of remaining oil activation and improvement in interlayer heterogeneity. The experimental results are shown in Figure 7 and Table 4.

From Table 4 and Figure 7, during the water flooding phase in the rhythmically designed interlayer heterogeneous model, the water cut in the high-permeability layer increased rapidly. The total oil recovery rate of the water flooding phase was 32.77%, with the middle- and high-permeability layers being the main contributors, at 12.70% and 15.16% respectively, while the relatively low-permeability layer was almost uninvaded, with only a 4.51% recovery rate. After the injection of the 0.3 PV nano-MoS_2_ system and a 12 h static period, the total oil recovery rate in the nano-MoS_2_ phase was 6.45%, mainly through the high-permeability layer at 3.59%, followed by the middle- and relatively low-permeability layers at 1.84% and 1.02%, respectively. This indicated that due to strong heterogeneity, the nano-MoS_2_ mainly flowed through high-permeability layer channels, exerting a wedging permeation effect, stripping oil films, and further activating remaining oil in the high-permeability layer water flooding, with a lower impact on middle- and relatively low-permeability layers. After the injection of 0.2 PV starch gel and a 72 h static period, followed by another 0.3 PV nano-MoS_2_ injection, the pressure increased sharply. The total oil recovery rate in the combination displacement phase was 12.20%, mainly through the middle- and low-permeability layers at 5.64% and 4.51%, with a significant decrease in water cut, while the high-permeability layer contributed 2.05%. This showed that starch gel effectively plugged high-permeability layers and preferential channels, forcing the subsequent nano-MoS_2_ solution to enter middle- and relatively low-permeability layers and secondary channels, effectively mobilizing oil in unswept reservoirs and significantly enhancing oil displacement efficiency.

In summary, the patterns of oil recovery rate changes during the displacement phases in both intra- and interlayer heterogeneity models were similar. During primary water flooding, the injection water had a limited impact on the uppermost low-permeability layer. When the nano-MoS_2_ system was injected alone, it primarily flowed through high-permeability channels, mainly further activating remaining oil films after high-permeability layer water flooding. When the starch gel system was injected and blocked these channels, it effectively plugged high-permeability channels, forcing subsequent nano-MoS_2_ towards middle- and relatively low-permeability layers, significantly enhancing oil recovery and showing a significant synergistic enhancement in oil displacement efficiency.

## 3. Conclusions

For the Henan Oilfield’s high-temperature heterogeneous reservoirs, laboratory experiments demonstrated the significant application potential of the combination displacement system of starch gel and nano-MoS_2_. The following results and conclusions were obtained.

1. The combination system comprising starch gel and nano-molybdenum disulfide exhibits notable thermal stability. At 95 °C, the starch gel consistently forms a rigid gel. Concurrently, nano-MoS_2_ exhibits significant capabilities in reducing interfacial tension and effectively emulsifying crude oil.
2. Under high-temperature (95 °C) heterogeneous reservoir conditions, the injection of 0.2 PV starch gel, followed by 0.3 PV of a 0.005% mass concentration nano-MoS_2_ solution, effectively plugged dominant channels. This forced the nano-MoS_2_ towards uninvaded areas, simultaneously enhancing sweep efficiency and displacement efficiency and significantly improving oil recovery by over 10 percentage points.
3. This study indicates that this combination displacement technology holds promise for enhancing oil recovery in high-temperature heterogeneous reservoirs of the Henan Oilfield, providing foundational support for further field applications.

## 4. Materials and Methods

### 4.1. Experimental Materials and Instruments

The main study area, H3IV2 layer of Henan Oilfield Anpeng block (Henan, China), provided formation water and crude oil (viscosity of 3.04 mPa·s). The pH of the formation water from the H3IV2 layer is 8.3, indicating it is slightly alkaline. The total salinity is 7755.66 mg/L, with a chloride ion content of 2512.25 mg/L, and the water type is NaHCO_3_ type. The ion composition of the H3IV2 layer formation water is provided by Henan Oilfield, as shown in Table 5.

Outcrop cores, 10 cm in length and 2.5 cm in diameter, with air-measured permeabilities of 100, 200, 300, and 500 mD, were used. The dimensions of the interlayer heterogeneous model were 30 × 30 × 6 cm, with permeability combinations of 80/200/400 mD and uniform layer thicknesses of 2 cm. Similarly, the intralayer heterogeneous model was of the same dimensions and permeability combinations. The nano-MoS_2_ concentrate (1% mass concentration, self-developed) and the modified starch were obtained from Henan Hengrui Starch Technology Co., Ltd (Luohe, China). Acrylamide (AM, 98%), N, N′-methylene bisacrylamide (crosslinker), and potassium persulfate (initiating agents) were analytical grade and purchased from Shanghai Macklin Biochemical Co., Ltd (Shanghai, China).

The experimental apparatus primarily included the following: interfacial tensiometer (SVT20N, Dataphysics, Filderstadt, Germany), pressure monitoring equipment, a high-temperature constant temperature oven (Jiangsu Hai‘an Petroleum Instrument Co., Ltd, Nantong, China ), an electronic balance, a vacuum pump, a double-cylinder piston pump (ISCO, Teledyne, Lincoln, NE, USA), a confining pressure hand pump and a core holder, a high-speed disperser (T 18 digital ULTRA-TURRAX, IKA, Staufen, Germany), among others.

### 4.2. Experimental Methods

#### 4.2.1. Preparation and Evaluation of Basic Properties of Starch Gel and Nano-MoS_2_ Solution

The MoS_2_ nanosheets were synthesized as reported in previous work [41,44,45,46]. Formation water from the H3IV2 layer of Anpeng and the 1% mass concentration nano-MoS_2_ concentrate were used to prepare a 0.005% mass concentration nano-MoS_2_ solution. The solution was subjected to ultrasonic oscillation for 15 min post-preparation, ensuring uniform and stable dispersion of nano-MoS_2_ in the formation water.

Interfacial tension between nano-MoS_2_ solution and the crude oil from layer H3IV2 in the Anpeng main area of the Henan Oilfield was determined at 95 °C using a rotating drop interfacial tensiometer.

Different oil–water ratio emulsions were prepared by mixing crude oil from layer H3IV2 in the Anpeng area with a 0.005% mass concentration nano-MoS_2_ solution (oil–water ratios of 3:7, 4:6, 5:5, 7:3) using a high-speed disperser (3000 r/min) for 3 min. The emulsions were then placed in a constant-temperature chamber at 95 °C, and the process of oil and water separation was observed and recorded; the separation rate was calculated. Microscopic images of the initial emulsion were captured using a microscope.

The starch gel was synthesized by modified starch, acrylamide (AM), a crosslinking agent, and initiator. Modified starch serves as a rigid framework within the starch gel structure. AM (acrylamide) is a typical monomer and acts as a flexible side chain within the starch gel structure. Crosslinkers can control the rate and extent of crosslinking reactions, thus influencing the strength of the gel system. Potassium persulfate can control the polymerization reaction rate, thereby regulating the gelation time.

The starch gel system is composed of 40,000 mg/L modified starch, 40,000 mg/L acrylamide, 1000 mg/L crosslinker, and 100 mg/L initiating agents.

Modified starch, acrylamide monomer, N, N′-methylenebisacrylamide, and potassium persulfate were weighed and slowly poured into deionized water with continuous stirring until complete dissolution. The stirring continued for 4 h to yield the experimental starch gel solution. The starch gel solution was placed in a constant-temperature oven set at 95 °C, using Sydansk’s gel strength code (GSC) [49,50] for the gelation time and gel strength of the starch gel solution.

#### 4.2.2. Evaluation of Oil Displacement Efficiency of Nano-MoS_2_ Solution in Cores

Basic parameters of the cores were measured, followed by vacuum saturation with water to calculate pore volume and water-measured permeability. The cores were then saturated with oil and aged for 7 days, with initial oil saturation recorded. Primary water flooding was conducted at a constant rate until the water cut reached 98%, followed by the injection of 0.3 PV nano-MoS_2_ oil displacement system. Subsequent water flooding continued until the water cut again reached 98%.

#### 4.2.3. Evaluation of Oil Displacement Efficiency of Starch Gel and Nano-MoS_2_ in Parallel Cores

After measuring basic core parameters, vacuum and saturating with water, the pore volume and water-measured permeability were calculated. The cores were oil-saturated and aged for 7 days, with initial oil saturation noted. Different permeability cores were paralleled, and primary water flooding was conducted at a constant rate until the water cut reached 98%. This was followed by nano-MoS_2_ displacement, injecting 0.3 PV nano-MoS_2_ oil displacement system and waiting for 12 h before continuing subsequent water flooding until the water cut reached 98%. Finally, a combination displacement with starch gel and nano-MoS_2_ was carried out, injecting 0.2 PV starch gel system and allowing it to sit for 72 h. Then, 0.3 PV nano-MoS_2_ oil displacement system was injected and left for 12 h before continuing with subsequent water flooding until the water cut reached 98%.

#### 4.2.4. Evaluation of Oil Displacement Efficiency of Starch Gel and Nano-MoS_2_ in Heterogeneity Models

**Figure 8:**
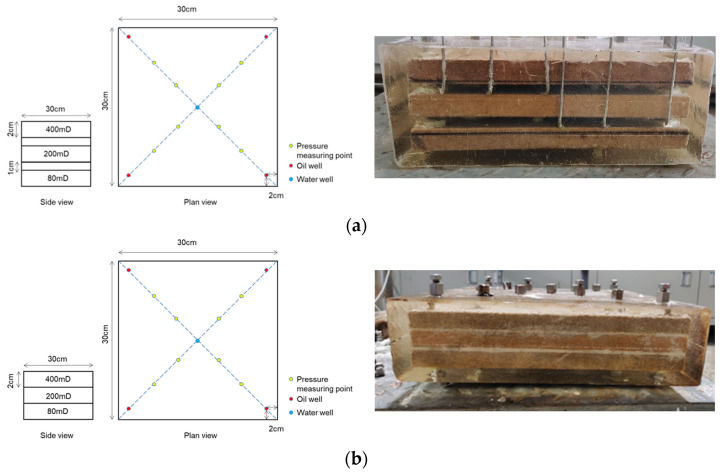
Schematic and physical models of interlayer (**a**); and intralayer (**b**) heterogeneity.

The intralayer and interlayer heterogeneous models, geometrically and physically similar to the main reservoirs of H3IV2 layer in Henan Oilfield Anpeng, were utilized (as shown in Figure 8). The interlayer heterogeneity model represents variations in permeability between different layers. This model comprises three independent rock cores, each with a thickness of 2 cm and separated by a 1 cm gap, with designated permeabilities of 80, 200, and 400 mD, respectively. The intralayer heterogeneity refers to variations in reservoir properties within a single sand layer, featuring a gradient of permeability values (80, 200, and 400 mD) from bottom to top. This layer is formed by bonding three separate sub-layers with sand and cement, representing a single heterogeneous sand layer.

The models, measuring 30 × 30 × 6 cm with permeability combinations of 80/200/400 mD and layer thicknesses of 2 cm, were sealed with epoxy resin and designed with one injection well and four production wells.

According to the experimental method, at a reservoir temperature of 95 °C, oil displacement effectiveness experiments were conducted for both intralayer and interlayer heterogeneous models. The models were vacuum saturated with water and the pore volumes calculated. After oil saturation and a 7-day aging period, initial oil saturations were recorded. Primary water flooding was then carried out at a constant rate until the water cut reached 98%. This was followed by nano-MoS_2_ displacement, injecting 0.3 PV nano-MoS_2_ oil displacement system and waiting for 12 h before continuing with subsequent water flooding until the water cut reached 98%. Finally, a combination displacement with starch gel and nano-MoS_2_ was performed, injecting 0.2 PV starch gel system and allowing it to sit for 72 h, then injecting 0.3 PV nano-MoS_2_ oil displacement system and waiting for 12 h before continuing with subsequent water flooding until the water cut reached 98%.

In the interlayer heterogeneous model oil displacement experiments, the production volumes of oil and liquid from the four producing wells in each layer were recorded. In the five-spot intralayer heterogeneous model experiments, the total production volumes of oil and liquid from the four producing wells across the three layers were noted.

## Figures and Tables

**Table 1:** The oil displacement effect of nano-MoS_2_ in cores with different permeabilities.

Core ID	Gas Permeability (mD)	Initial Water Flooding Recovery Rate (%)	Final Recovery Rate (%)	Enhance Recovery Rate (%)
1	100	36.07	54.10	18.03
2	200	45.72	65.78	20.06
3	300	47.95	62.47	14.52
4	500	50.86	63.17	12.31

**Table 2:** The oil displacement effect of combination system in cores with different permeabilities.

Core ID	Gas Permeability (mD)	Initial Water Flooding Recovery Rate (%)	Nano-MoS_2_ Flooding Enhance Recovery Rate (%)	Combination Displacement Enhance Recovery Rate (%)	Final Recovery Rate (%)
3	100	37.47	5.90	4.72	48.10
	300	44.77	7.44	6.34	58.55
Whole		41.18	6.69	5.54	53.41
4	100	30.15	2.48	6.80	39.43
	500	46.63	7.80	5.84	60.27
Whole		38.58	5.20	6.31	50.09

**Table 3:** The oil displacement effectiveness in intralayer heterogeneous model.

Core ID	Gas Permeability (mD)	Initial Water Flooding Recovery Rate (%)	Nano-MoS_2_ Flooding Enhance Recovery Rate (%)	Combination Displacement Enhance Recovery Rate (%)	Final Recovery Rate (%)
5	80	27.47	5.07	15.33	47.87
	200				
	400				

**Table 4:** The oil displacement effectiveness in interlayer heterogeneous model.

Core ID	Gas Permeability (mD)	Initial Water Flooding Recovery Rate (%)	Nano-MoS_2_ Flooding Enhance Recovery Rate (%)	Combination Displacement Enhance Recovery Rate (%)	Final Recovery Rate (%)
6	80	15.16	3.59	2.05	21.82
	200	12.70	1.84	5.64	20.59
	400	4.51	1.02	4.51	10.04
Whole		32.77	6.45	12.20	52.45

**Table 5:** Ion composition of the formation water in H3IV2 formation, Henan Oilfield.

Ion(s)	HCO_3_^−^	Cl^−^	SO_4_^2−^	Ca^2+^	Mg^2+^	K^+^	Na^+^	Salinity
Concentration, mg/L	1476.68	2512.25	777.87	23.42	4.03	86.23	2524	7755.66

## Data Availability

All data and materials are available on request from the corresponding author. The data are not publicly available due to ongoing researches using a part of the data.

## References

[1] Wang L., Tian Y., Yu X., Wang C., Yao B., Wang S., Winterfeld P.H., Wang X., Yang Z., Wang Y. (2017). Advances in improved/enhanced oil recovery technologies for tight and shale reservoirs. Fuel.

[2] Yang Y., Yuan W., Hou J., You Z. (2022). Review on physical and chemical factors affecting fines migration in porous media. Water Res..

[3] Zheng J., Wang Z., Ju Y., Tian Y., Jin Y., Chang W. (2021). Visualization of water channeling and displacement diversion by polymer gel treatment in 3D printed heterogeneous porous media. J. Pet. Sci. Eng..

[4] Alajmi A.F., Gharbi R., Algharaib M. (2014). The effect of heterogeneity and well configuration on the performance of hot water flood. J. Pet. Sci. Eng..

[5] Yuan B., Wood D.A. (2018). A comprehensive review of formation damage during enhanced oil recovery. J. Pet. Sci. Eng..

[6] Kang W.-L., Zhou B.-B., Issakhov M., Gabdullin M. (2022). Advances in enhanced oil recovery technologies for low permeability reservoirs. Pet. Sci..

[7] Yu H., Wang L., Zhou D., Wang F., Li S., Li J., Chen X., Cao A., Han H. (2021). Experimental study on sweep characteristics of gas gravity drainage in the interlayer oil reservoir. Front. Energy Res..

[8] Tang H., Wen X., Zhang X., Ren X., Liu H. (2014). Water-oil displacing modeling experiment of interlayer heterogeneous conglomerate reservoir. J. Southwest Pet. Univ. Sci. Technol. Ed..

[9] Zhang W., Zhang J., Xie J. (2008). Research on reservoir bed heterogeneity, interlayers and seal layers and controlling factors of 2+ 3 sands of upper second member, Shahejie Formation, in the west of the Pucheng Oilfield. Pet. Sci..

[10] Jiang L., Zhao W., Zhang B., Zheng C., Hong F., Hao J. (2022). Characterization method of tight sandstone reservoir heterogeneity and tight gas accumulation mechanism, Jurassic Formation, Sichuan Basin, China. Geofluids.

[11] Li J., Wang Z., Yang H., Yang H., Wu T., Wu H., Wang H., Yu F., Jiang H. (2020). Compatibility evaluation of in-depth profile control agents in dominant channels of low-permeability reservoirs. J. Pet. Sci. Eng..

[12] Chen X., Li Y., Liu Z., Zhang J., Chen C., Ma M. (2020). Investigation on matching relationship and plugging mechanism of self-adaptive micro-gel (SMG) as a profile control and oil displacement agent. Powder Technol..

[13] Hua Z., Lin M., Guo J., Xu F., Li Z., Li M. (2013). Study on plugging performance of cross-linked polymer microspheres with reservoir pores. J. Pet. Sci. Eng..

[14] Yu B., Zhao S., Long Y., Bai B., Schuman T. (2022). Comprehensive evaluation of a high-temperature resistant re-crosslinkable preformed particle gel for water management. Fuel.

[15] Zhao G., Dai C., You Q. (2018). Characteristics and displacement mechanisms of the dispersed particle gel soft heterogeneous compound flooding system. Pet. Explor. Dev..

[16] Yin H., Yin X., Cao R., Zeng P., Wang J., Wu D., Luo X., Zhu Y., Zheng Z., Feng Y. (2021). In situ crosslinked weak gels with ultralong and tunable gelation times for improving oil recovery. Chem. Eng. J..

[17] Elsharafi M.O., Bai B. (2012). Effect of weak preformed particle gel on unswept oil zones/areas during conformance control treatments. Ind. Eng. Chem. Res..

[18] Hua Z., Lin M., Dong Z., Li M., Zhang G., Yang J. (2014). Study of deep profile control and oil displacement technologies with nanoscale polymer microspheres. J. Colloid Interface Sci..

[19] Abdulbaki M., Huh C., Sepehrnoori K., Delshad M., Varavei A. (2014). A critical review on use of polymer microgels for conformance control purposes. J. Pet. Sci. Eng..

[20] Zhang F., Hou B., Wang S., Chen H., Yang J., Fan H., Gui L. (2023). Study on the Profile Control and Oil Displacement Mechanism of a Polymer Nano-microsphere for Oilfield. Energy Fuels.

[21] Yongqiang T., Jirui H., Chenghui L. (2013). Water shut off in a horizontal well: Lab experiments with starch graft copolymer agent. J. Pet. Sci. Eng..

[22] Zhao F., Hao H., Hou J., Hou L., Song Z. (2015). CO_2_ mobility control and sweep efficiency improvement using starch gel or ethylenediamine in ultra-low permeability oil layers with different types of heterogeneity. J. Pet. Sci. Eng..

[23] Hao H., Hou J., Zhao F., Song Z., Hou L., Wang Z. (2016). Gas channeling control during CO_2_ immiscible flooding in 3D radial flow model with complex fractures and heterogeneity. J. Pet. Sci. Eng..

[24] Luo Q., Tang K., Bai L., Li K., Sun P., Xu C., Zhao Y., Zhu D. (2021). Development of in-situ starch grafted copolymerized gels for conglomerate reservoir conformance control and oil recovery improvement. J. Pet. Sci. Eng..

[25] Leng G., Yan W., Li F., Wang B., Yuan G., Chu C., Li Y., Gao C. (2023). Improved oil recovery in sandstone reservoirs with deep profile control technology: A comparative study between modified starch gel and polymer gel. Geoenergy Sci. Eng..

[26] Li W., Dai C., Jan O., Hafiz A., Tao J., He X., Zhao G. (2018). Adsorption and retention behaviors of heterogeneous combination flooding system composed of dispersed particle gel and surfactant. Colloid Surface A..

[27] Hadia N.J., Ng Y.H., Stubbs L.P., Torsæter O. (2021). High salinity and high temperature stable colloidal silica nanoparticles with wettability alteration ability for eor applications. Nanomaterials.

[28] Gizzatov A., Mashat A.A., Kosynkin D.V., Alhazza N., Kmetz A., Eichmann S.L., Abdel-Fattah A.I. (2019). Nanofluid of petroleum sulfonate nanocapsules for enhanced oil recovery in high-temperature and high-salinity reservoirs. Energy Fuels.

[29] Zhu D., Wei L., Wang B., Feng Y. (2014). Aqueous hybrids of silica nanoparticles and hydrophobically associating hydrolyzed polyacrylamide used for EOR in high-temperature and high-salinity reservoirs. Energies.

[30] Lu T., Li Z., Zhou Y., Zhang C. (2017). Enhanced oil recovery of low-permeability cores by SiO_2_ nanofluid. Energy Fuels.

[31] Murshed S.M.S., Tan S.H., Nguyen N.T. (2008). Temperature dependence of interfacial properties and viscosity of nanofluids for droplet-based microfluidics. J. Phys. D Appl. Phys..

[32] Rezvani H., Panahpoori D., Riazi M., Parsaei R., Tabaei M., Cortés F.B. (2020). A novel foam formulation by Al_2_O_3_/SiO_2_ nanoparticles for EOR applications: A mechanistic study. J. Mol. Liq..

[33] Bahraminejad H., Manshad A.K., Riazi M., Ali J.A., Sajadi S.M., Keshavarz A. (2019). CuO/TiO_2_/PAM as a novel introduced hybrid agent for water—Oil interfacial tension and wettability optimization in chemical enhanced oil recovery. Energy Fuels.

[34] Alnarabiji M.S., Yahya N., Nadeem S., Adil M., Baig M.K., Ben Ghanem O., Azizi K., Ahmed S., Maulianda B., Klemeš J.J. (2018). Nanofluid enhanced oil recovery using induced ZnO nanocrystals by electromagnetic energy: Viscosity increment. Fuel.

[35] AfzaliTabar M., Alaei M., Bazmi M., Khojasteh R.R., Koolivand-Salooki M., Motiee F., Rashidi A. (2017). Facile and economical preparation method of nanoporous graphene/silica nanohybrid and evaluation of its Pickering emulsion properties for Chemical Enhanced oil Recovery (C-EOR). Fuel.

[36] Esmaeilzadeh P., Hosseinpour N., Bahramian A., Fakhroueian Z., Arya S. (2014). Effect of ZrO_2_ nanoparticles on the interfacial behavior of surfactant solutions at air–water and n-heptane–water interfaces. Fluid Phase Equilibria.

[37] Hill D., Barron A.R., Alexander S. (2020). Controlling the wettability of plastic by thermally embedding coated aluminium oxide nanoparticles into the surface. J. Colloid Interface Sci..

[38] Alnarabiji M.S., Husein M.M. (2020). Application of bare nanoparticle-based nanofluids in enhanced oil recovery. Fuel.

[39] Zhang H., Ramakrishnan T.S., Nikolov A., Wasan D. (2016). Enhanced oil recovery driven by nanofilm structural disjoining pressure: Flooding experiments and microvisualization. Energy Fuels.

[40] Patel H., Shah S., Ahmed R., Ucan S. (2018). Effects of nanoparticles and temperature on heavy oil viscosity. J. Pet. Sci. Eng..

[41] Raj I., Qu M., Xiao L., Hou J., Li Y., Liang T., Yang T., Zhao M. (2019). Ultralow concentration of molybdenum disulfide nanosheets for enhanced oil recovery. Fuel.

[42] Wei P., Luo Q., Edgehouse K.J., Hemmingsen C.M., Rodier B.J., Pentzer E.B. (2018). 2D particles at fluid–fluid interfaces: Assembly and templating of hybrid structures for advanced applications. ACS Appl. Mater. Interfaces.

[43] Nonomura Y., Komura S., Tsujii K. (2004). Adsorption of disk-shaped Janus beads at liquid–liquid interfaces. Langmuir.

[44] Qu M., Hou J., Liang T., Qi P. (2021). Amphiphilic rhamnolipid molybdenum disulfide nanosheets for oil recovery. ACS Appl. Nano Mater..

[45] Liang T., Hou J., Xi J. (2023). Mechanisms of nanofluid based modification MoS_2_ nanosheet for enhanced oil recovery in terms of interfacial tension, wettability alteration and emulsion stability. J. Dispers. Sci. Technol..

[46] Qu M., Liang T., Hou J., Liu Z., Yang E., Liu X. (2022). Laboratory study and field application of amphiphilic molybdenum disulfide nanosheets for enhanced oil recovery. J. Pet. Sci. Eng..

[47] Liang T., Yang C., Zhang Y., Li P., Qu M., Hou J. (2023). Research and Application Progress of Nanofluid for Enhanced Oil Recovery. Xinjiang Oil Gas.

[48] Xu H., Li Y., Yu G.-L., Yang S.-S., Li B.-J., Zhou F.-J., Yao E.-D., Bai H., Liu Z.-Y. (2023). The enhancement of performance and imbibition effect of slickwater-based fracturing fluid by using MoS_2_ nanosheets. Pet. Sci..

[49] Sydansk R.D., Argabright P.A. (1987). Conformance Improvement in a Subterranean Hydrocarbon-Bearing Formation Using a Polymer Gel. U.S. Patent.

[50] Sydansk R.D. (1990). A newly developed chromium (lll) gel technology. SPE Reserv. Eng..

[51] Motraghi F., Manshad A.K., Akbari M., Ali J.A., Sajadi S.M., Iglauer S., Keshavarz A. (2023). Interfacial tension reduction of hybrid crude-oil/mutual-solvent systems under the influence of water salinity, temperature and green SiO_2_/KCl/Xanthan nanocomposites. Fuel.

[52] Hassan M.E., Nielsen R.F., Calhoun J.C. (1953). Effect of pressure and temperature on oil-water interfacial tensions for a series of hydrocarbons. J. Pet. Technol..

[53] Gaonkar A.G. (1992). Effects of salt, temperature, and surfactants on the interfacial tension behavior of a vegetable oil/water system. J. Colloid Interf. Sci..

[54] Arab D., Kantzas A., Bryant S.L. (2018). Nanoparticle stabilized oil in water emulsions: A critical review. J. Pet. Sci. Eng..

[55] Stancik E.J., Kouhkan M., Fuller G.G. (2004). Coalescence of particle-laden fluid interfaces. Langmuir.

[56] Simovic S., Prestidge C.A. (2004). Nanoparticles of varying hydrophobicity at the emulsion droplet−water interface: Adsorption and coalescence stability. Langmuir.

[57] Zhu D.-Y., Zhao Y.-H., Zhang H.-J., Zhao Q., Shi C.-Y., Qin J.-H., Su Z.-H., Wang G.-Q., Liu Y., Hou J.-R. (2023). Combined imbibition system with black nanosheet and low-salinity water for improving oil recovery in tight sandstone reservoirs. Pet. Sci..

[58] Guillen V.R., Carvalho M.S., Alvarado V. (2012). Pore scale and macroscopic displacement mechanisms in emulsion flooding. Transport Porous Med..

[59] Ding M.-C., Li Q., Yuan Y.-J., Wang Y.-F., Zhao N., Han Y.-G. (2022). Permeability and heterogeneity adaptability of surfactant-alternating-gas foam for recovering oil from low-permeability reservoirs. Pet. Sci..

[60] Gong H., Zhang H., Xu L., Li K., Yu L., Li Y., Dong M. (2017). Further enhanced oil recovery by branched-preformed particle gel/HPAM/surfactant mixed solutions after polymer flooding in parallel-sandpack models. RSC Adv..

[61] Le N.N.H., Sugai Y., Vo-Thanh H., Nguele R., Ssebadduka R., Wei N. (2022). Experimental investigation on plugging performance of CO_2_ microbubbles in porous media. J. Pet. Sci. Eng..

